# The prognostic impact of lung adenocarcinoma predominance classification relating to pathological factors in lobectomy, the Japanese Joint Committee of Lung Cancer Registry Database in 2010

**DOI:** 10.1186/s12885-022-09973-8

**Published:** 2022-08-10

**Authors:** Hiroyuki Ito, Hiroshi Date, Yasushi Shintani, Etsuo Miyaoka, Ryoichi Nakanishi, Mitsutaka Kadokura, Shunsuke Endo, Masayuki Chida, Ichiro Yoshino, Hidemi Suzuki, the Japanese Joint Committee of Lung Cancer Registry

**Affiliations:** 1 Department of Thoracic Surgery, Kanagawa Cancer Center, 2-3-2 Nakao, Asahi-ku, Yokohama, 241-8515 Japan; 2 Department of Thoracic Surgery, Kyoto University Graduate School of Medicine, Kyoto, Japan; 3 Department of General Thoracic Surgery, Osaka University Graduate School of Medicine, Osaka, Japan; 4 Department of Mathematics, Tokyo University of Science, Tokyo, Japan; 5 Department of Oncology, Immunology and Surgery, Nagoya City University Graduate School of Medical Sciences, Nagoya, Japan; 6 Respiratory Disease Center, Showa University Northern Yokohama Hospital, Yokohama-shi, Japan; 7 Department of Thoracic Surgery, Jichi Medical School, Shimotsuke, Japan; 8 Department of General Thoracic Surgery, Dokkyo Medical University, Shimotsuga-gun, Japan; 9 Department of General Thoracic Surgery, Graduate School of Medicine, Chiba University, Chiba, Japan

**Keywords:** Adenocarcinoma, Adenocarcinoma predominance, Lobectomy, The Japanese lung cancer registry

## Abstract

**Objective:**

We studied the prognosis and clinicopathological background of lung adenocarcinoma predominance among patients who underwent lobectomy using data from the Japanese Joint Committee of Lung Cancer Registry.

**Methods:**

Two thousand eight hundred sixty-three cases were extracted. Recurrence free survival (RFS) rates, overall survival (OS) rates and clinicopathological factors and epidermal growth factor receptor (EGFR) mutation status were examined.

**Results:**

Median follow-up period was 65.5 months. Adenocarcinoma predominance was sub-grouped according to OS and RFS rate. In pathological stage I, 5-year RFS and OS rates were respectively 92.2% and 95.8% in group A (adenocarcinoma-in-situ + minimally invasive adenocarcinoma), 89.3% and 92.1% in group B (lepidic), 79.2% and 89.7% in group C (papillary + acinar + variants) and 69.0% and 79.0% in group D (solid + micropapillary). In pathological stage II + IIIA, they were, 43.6% and 72.4% in B, 39.5% and 66.9% in C and 31.0% and 53.7% in D. Group D showed significant worst outcome both in stage I and II + IIIA. Up stage rate from clinical stage I to pathological stage II + IIIA was 0.0%, 3.7%, 15.9% and 33.3%. The frequency of lymph-vessel, vascular, pleura invasion and positive EGFR mutation were 0.0%, 0.0%, 0.0% and 57.1% in group A, 15.6%, 10.0%, 12.1% and 55.1% in B, 36.6%, 31.8%, 29.7% and 44.9% in C, 50.2%, 57.8%, 38.9% and 21.3% in D. In group D, lymph-vessel, vascular and pleura invasion were most, EGFR mutation was least frequent not only in pathological stage I but also stage II + IIIA. In multivariate analysis, age, pathological stage, vascular invasion, and group D were independent factors affected RFS and OS.

**Conclusion:**

Limited to lobectomy cases, solid + micropapillary was independent prognostic factor both in early and locally advanced stage. Its malignant degree was related to the frequency of pathological invasive factors and EGFR mutation status.

## Introduction

Adenocarcinoma is the most frequent histological type of lung cancer, and its frequency has been increasing [1, 2]. In 2011, lung adenocarcinoma predominant subtype classifications were proposed by the International Association for the Study of Lung Cancer, the American Thoracic Society, and the European Respiratory Society (IASLC/ATS/ERS) [3]. At that time, adenocarcinoma was categorized into 4 types: preinvasive lesions of adenocarcinoma in situ (AIS), minimally invasive adenocarcinoma (MIA, ≤ 3 cm predominant lepidic growth within 5 mm invasion), invasive adenocarcinoma and variants. Invasive adenocarcinoma was subcategorized into 5 subtypes (lepidic, papillary, acinar, solid and micropapillary). AIS and MIA were reported to show excellent outcome [3]. Other than AIS and MIA, the surgical outcome of invasive adenocarcinoma and variants vary, and the predominant subtype of invasive adenocarcinoma has been reported to be one of the prognostic factors [4–14]. However, these findings were based on a small number of cases from a limited number of facilities lacking uniform surgical procedures, a narrow range of pathological stages, and non-uniform backgrounds. Thus, as objective prognostic information, the data is considered insufficient. The database of completely resected NSCLC cases compiled by the Japanese Joint Committee of Lung Cancer Registry (JJCLCR) in 2010 consists of comprehensive patent background information, surgical procedures, pathological information including adenocarcinoma predominance and long-term prognostic outcome [2]. Using this nationwide database and focusing only on patients who underwent lobectomy with mediastinal lymph-node dissection to exclude the bias of surgical procedure, we herein analyzed the lung adenocarcinoma predominance for prognostic impact after surgery and the relation of clinicopathological factors.

## Methods

### Registry [2]

The JJCLCR conducted a nationwide registry study of patients who had undergone lung cancer surgery. The committee asked 629 teaching hospitals certified by the Japanese Board of General Thoracic Surgery to participate in the study. The registry followed the ethical guidelines for epidemiologic studies; the study protocol was approved by the institutional review board of Osaka University Hospital (approval No. 15321) and registered at University Hospital Medical Information Network- Clinical Trials Registry (UMIN000020215).

### Patients

From the 18,973 cases in the JJCLR dataset in 2010, cases of a lobectomy with mediastinal lymph-node dissection achieving R0 with comprehensive information of adenocarcinoma predominance by IASLC/ATS/ERS classification were selected. Cases of induction therapy, histology other than adenocarcinoma, did not meet the pathological definition of IASLC / ATS / ERS classification and a final pathological stage higher than IIIA were excluded. The final number of cases analyzed for this study was 2,863.

Overall survival (OS) and recurrence-free survival (RFS) rates were calculated, and their relationship to the following clinicopathological factors was examined: age, gender, smoking history, operation time, transfusion, pathological stage according to TNM 7^th^ Edition classification, lymphatic vessel invasion, vascular invasion, pleural invasion, epidermal growth factor receptor (EGFR) mutation status, adjuvant therapy, clinical stage, pathological stage and lung adenocarcinoma predominant histological subtype. Predominant subtypes of adenocarcinoma were pathologically diagnosed at each facility, not undergone by a central review. Variants consisted of invasive mucinous, colloid, fetal (low and high grade) and enteric adenocarcinoma. Postoperative recurrence was determined by each physician and histopathologic confirmation at the time of recurrence was not required.

### Statistical analysis

OS was defined as the time interval from surgery to death from any cause. RFS was defined as the time interval from surgery to the time of first recurrence or death. For those patients who were living, data on RFS were censored at the last visit.

The data were analyzed using the Kaplan–Meier method and compared using the log-rank test. Patients were excluded from the analyses for RFS if the time of their recurrence was not available. Clinicopathological data were evaluated from univariate analysis. Descriptive statistics used included means and standard deviations for continuous variables and percentages for categorical variables, which were compared using Mann–Whitney U and chi-squared tests. Multivariate analysis was performed with a Cox proportional-hazards regression model. Results are summarized as a hazard ratio (HR) with 95% confidence intervals (CI). HR represents the relative increase (or decrease if < 1) in the risk of recurrence or death. *P* values of less than 0.05 were considered to indicate statistical significance.

## Results

The median follow-up period was 65.5 months. Table 1 shows the background characteristics of the 2,863 patients. The mean age was 67.0 years, and the gender ratio was nearly equivalent. More than half of the cases had a history of smoking. In clinical stage. 86.4% of all cases were stage I, stage IIIA was only 3.8%. According to the IASLC/ATS/ERS classification, AIS and MIA accounted for 10.8% of all cases and the most common, invasive adenocarcinoma, accounted for 87.5%. The proportion of variants was small (1.6%). The most frequent type of invasive adenocarcinoma was papillary predominant (40.6%), followed by acinar (18.8%) and lepidic (18.2%). Solid accounted for 7.8% and micropapillary was only 2.2%. 86.4% of all cases was clinical stage I. According to the pathological stage, stage IA was the most frequent (49.8%) followed by stage IB (25.6%). In total, 75.4% of all cases was pathological stage I. The rate for pathological stage II was 12.9% and stage IIIA was 11.0%.

**Table 1:** Patient characteristics

n	2,863
Age, mean ± SD	66.71 ± 9.149
Sex (male), n (%)	1,478 (51.6%)
Smoking history, n (%)	1,471 / 2,779 (52.9%)
Procedure, n (%)	
Lobectomy	2,863 (100%)
Operation time (min.), mean ± SD	205.3 ± 66.89
Histological subtype, n (%)	
AIS	135 (4.7%)
MIA	175 (6.1%)
Invasive adenocarcinoma	2,506 (87.5%)
Lepidic	521 (18.2%)
Acinar	537 (18.8%)
Papillary	1,161 (40.6%)
Micropapillary	63 (2.2%)
Solid	224 (7.8%)
Variants	47 (1.6%)
Invasion	
Lymph vessel invasion ( ±), n (%)	777 / 1,785 (30.3%)
Vascular invasion ( ±), n (%)	695 / 1,875 (27.0%)
Pleural invasion ( ±), n (%)	691 / 2,156 (24.3%)
EGFR status ( ±), n (%)	641 / 783 (45.0%)
Adjuvant chemotherapy ( ±), n (%)	1,017 / 1,820 (35.8%)
Clinical stage, n (%)	
Stage IA	1732 (60.5%)
Stage IB	749 (26.2%)
Stage IIA	190 (6.6%)
Stage IIB	83 (2.9%)
Stage IIIA	98 (3.4%)
Pathological stage, n (%)	
Stage IA	1,426 (49.8%)
Stage IB	732 (25.6%)
Stage IIA	260 (9.1%)
Stage IIB	110 (3.8%)
Stage IIIA	314 (11.0%)

*AIS* Adenocarcinoma in situ, *MIA* Minimally invasive adenocarcinoma, *SD* standard deviation, Min Minutes

EGFR mutation was examined in 1424 case: 49.7% of all cases. The frequency of positive EGFR mutation was 45.0% for all cases examined. 912 cases were examined in no recurrence (40.8%), but 551 cases were in recurred cases (79.7%). The number of no examination of EGFR mutation was 1441 cases, 90.3% in no recurrence and 9.7% in recurred cases. Examination of EGFR mutation was frequently performed in recurred cases (*p* < 0.001).

35.8% of all cases had received adjuvant chemotherapy.

### Recurrence free survival

**Fig. 1:**
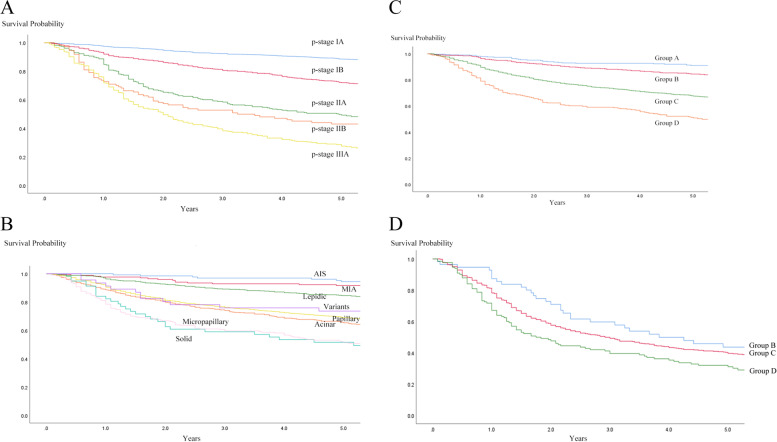
**A**. RFS curves according to pathological stage. The 5-year RFS rates was 88.5% in patients with stage IA, 72.0% in stage IB, 49.2% in stage IIA, 43.0% in stage IIB and 27.5% in stage IIIA. **B**. RFS curves according to histologic predominance. The 5-year RFS rates was 94.5% in patients with AIS, 91.7% in MIA, 84.5% in lepidic, 73.6% in variants, 69.0% in papillary, 65.3% in acinar, 51.6% in micropapillary, and 51.4% in solid. **C** RFS curves for each histological groups in pathological stage I. The 5-year RFS rate was 92.2% in patients with group A (AIS + MIA), 89.3% in group B (lepidic), 79.2% in group C (papillary + acinar + variants) and 69.0% in group D (solid + micropapillary). **D** RFS curves of each histological groups in pathological stage II + IIIA. The 5-year RFS rates was 43.6% in patients with group B, 39.5% in group C and 31.0% in group D

The RFS rate was 72.1% at 5 years in all cases. The RFS curves according to pathological stage are shown in Fig. 1A. Each stage showed significant difference to a higher stage (IA vs. IB; *p* < 0.001, IB vs. IIA; *p* < 0.001, IIA vs. IIIA; *p* < 0.001, IIB vs. IIIA; *p* = 0.013). For additional analysis, the pathological stages were combined and termed as I (IA + IB) and II + IIIA (IIA + IIB + IIIA). The 5-year RFS rate for stage I was 82.7% and 38.0% for stage II + IIIA.

RFS curves according to predominant histological subtype are shown in Fig. 1B. AIS and MIA showed similar RFS curves (*p* = 0.446). Papillary, acinar and variants showed similar RFS curves; no statistical differences were observed (acinar vs. papillary; *p* = 0.209, acinar vs. variants; *p* = 0.65, papillary vs. variants; *p* = 0.978). RFS curves for solid and micropapillary almost overlapped (*p* = 0.823). Therefore, adenocarcinoma predominant subtypes can be combined to AIS + MIA (group A), lepidic (group B), papillary + acinar + variants (group C) and solid + micropapillary (group D). The 5-year RFS rate of group C was 67.5% and 50.8% of group D.

In pathological stage I, the RFS curves for each predominant subtype groups are shown in Fig. 1C. The RFS curve for group A almost overlapped with group B (*p* = 0.171). Group C showed worse RFS than Group A (*p* < 0.001, HR [95%CI] = 2.720 [1.841–4.011]) and group B (*p* < 0.001, HR = 1.987 [1.483–2.660]). Group D showed worse RFS than group C (*p* = 0.003, HR = 1.571 [1.159 – 2.151]).

In pathological stage II + IIIA, RFS curves are shown in Fig. 1D. The difference between group B and C was not observed (*p* = 0.181). Group D showed significant worse outcome than group B (*p* = 0.008, HR = 1.678 [1.109–2.538]) and group C (*p* = 0.012, HR = 1.338 [1.064–1.678]).

### Overall survival

**Fig. 2:**
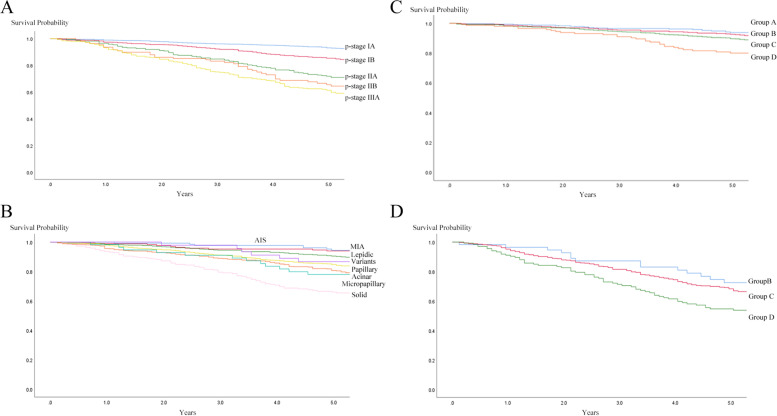
**A**. OS curves according to pathological stage. 5-year OS rates were 92.9% in patients with stage IA, 85.3% in stage IB, 70.9% in p stage IIA, 64.4% in stage IIB and 59.8% in stage IIIA. **B**. OS curves according to predominant histological subtype. The 5-year OS rates was 94.4% in patients with AIS. 94.0% in MIA, 90.2% in lepidic, 86.7% in variants, 84.1% in papillary, 80.4% in acinar, 78.0% in micropapillary and 65.4% in solid. **C**. OS curves according to histological groups in pathological stage I. The 5-year OS rates was 95.8% in patients with group A, 92.1% in group B, 89.7% in group C and 79.0% in group D. **D**. OS curves according to histological groups in pathological stage II + III. The 5-year OS rates was 72.4% in patients with group B, 66.9% in group C and 53.7% in group D

The OS rate at 5 years was 84.1% in all cases. OS curves according to pathological stage are shown in Fig. 2A. Stage IA showed significant better outcome than that of higher stages (*p* < 0.001, all), and stage IB also showed better outcome then higher stages (*p* < 0.001, all). But the differences between IIA and IIB, IIB and IIIA were not significant (*p* = 0.419, *p* = 0.161). From these results, pathological stages can be combined and indicated as stage IA + IB and IIA + IIB + IIIA. The 5-year OS rates were 90.3% for stage IA + IB and 64.6% for stage IIA + IIB + IIIA.

The OS curves according to predominant histological subtype are shown in Fig. 2B. The statistical difference between AIS, MIA and lepidic were not observed (*p* = 0.634, 0.107, 0.231). The difference between acinar and variants, papillary and variants were not observed (*p* = 0.593, *p* = 0.830). Solid showed worse outcome than that of AIS, MIA, lepidic, acinar, and papillary (*p* < 0.001, all), but not with micropapillary (*p* = 0.119). Thus, these predominant histological subtypes can be combined in the same manner as for RFS.

In pathological stage I, OS curves of histological subtype groups are shown in Fig. 2C. Group A almost overlapped with group B (*p* = 0.561). Group C showed worse outcome than that of group A (*p* = 0.018, HR = 1.691 [1.093–2.630]), and group B (*p* = 0.039, HR = 1.448 [1.028–2.095]). Group D showed worse outcome than group C (*p* = 0.002, HR = 1.878 [1.265–2.779]).

The OS curves in pathological stage II + III are shown in Fig. 2D. No significant difference was observed among group B and C. Group D showed significant worse outcome than group B (*p* = 0.018, HR = 1.949 [1.116–3.430]) and group C (*p* = 0.005, HR = 1.498 [1.12–1.982]).

### Patient characteristics for predominant histological subtype groups in all stages, stage I and II + IIIA

Table 2 shows the characteristics of patients with the predominant subtype groups in all pathological stages. For group D, male and smoking history, positive lymphatic vessel invasion (Ly +), vascular invasion (V +) and pleura invasion (PL +) were most frequent, group C was secondary frequent.

**Table 2:** Patient information for predominant histological subtypes in all cases

		Total	Group A (AIS + MIA)	Group B (Lepidic)	Group C (Papillary + acinar + variant)	Group D (Solid + micropapillary)	*p*-value
n		2863	304	526	1745	287	
Sex (male/Female), n (%)		1478/1385 (51.6%)	119/185 (39.1%)	233/293 (44.3%)	940/805 (53.8%)	185/102 (64.4%)	< 0.001
Age, mean ± SD		66.71 ± 9.15	66.49 ± 9.67	67.72 ± 8.20	66.46 ± 9.23	65.88 ± 9.38	0.025
Smoking history ( ±), n (%)		1471 / 1308 (52.9%)	110/187 (37.0%)	222 / 292 (43.2%)	741 / 594 (55.5%)	205 / 72 (74.0%)	< 0.001
Operation time (min.), mean ± SD		205.25 ± 66.89	203.47 ± 62.70	198.60 ± 66.50	207.41 ± 67.76	204.13 ± 66.12	0.017
Invasion, n (%)							
	Lymph vessel invasion ( ±), n (%)	777 / 1785 (30.3%)	0 / 273 (0%)	76 / 412 (15.6%)	565 / 978 (36.6%)	131 / 130 (50.2%)	< 0.001
	Vascular invasion ( ±), n (%)	695 / 1875 (27.3%)	0 / 278 (0%)	49 / 440 (10.0%)	494 / 1059 (31.8%)	152 / 111 (57.8%)	< 0.001
	Pleural invasion ( ±), n (%)	691 / 2156 (24.3%)	0 / 316 (0%)	63/ 461 (12.1%)	517 / 1224 (29.7%)	111 / 174 (38.9%)	< 0.001
EGFR mutation ( ±)		641 / 783 (45.0%)	72 / 54 (57.1%)	118 / 96 (55.1%)	426 / 522 (44.9%)	33 / 122 (21.3%)	< 0.001
Adjuvant therapy ( ±)		1017 / 1820 (35.8%)	17 / 287 (5.9%)	152 / 368 (29.2%)	697 / 1034 (40.3%)	143 / 140 (50.5%)	< 0.001
Clinical stage IA / IB / IIA / IIB / IIIA		1732 / 749 / 190 / 83 / 98	272 / 31 / 1 / 0 / 0	316 / 171 / 22 / 9 / 8	1015 / 462 / 132 / 59 / 77	143 / 82 / 32 / 11 / 19	< 0.001
Pathological stage IA / IB / IIA / IIB / IIIA		1426 / 732 / 260 / 110 / 314	298 / 6 / 0 / 0 / 0	319 / 150 / 18 / 7 / 32	749 / 493 / 195 / 75 / 233	82 / 68 / 50 / 23 / 64	< 0.001

In clinical stage, stage I was less frequent of 78.4% (225/287) in group D, followed by 84.6% (1477/1745) of group C, 92.6% (487/526) of group B and 99.7% of group A (303/304) (*p* < 0.01). This was similar in pathological stage; 52.3% (150/287), 71.1% (1242/1745), 89.2% (469/526) and 100% (304/304) (*p* < 0.01). Concerning pathological stage II + IIIA, group D was most frequent of 47.7% followed by 28.8% of group C, 10.8% of group B (*p* < 0.01). Up stage rate from clinical stage I to pathological stage II + IIIA in each group was 0.0%, 3.7%, 15.9% and 33.3%. EGFR mutation in group D was least frequent o21.3% than 51.3% of other groups (*p* < 0.01).

Table 3 shows the characteristics in pathological stage I. For group D, male and smoking history, Ly + , V + and PL + were most frequent. In group B, the frequency of these factors was intermediate between group A and C. When group C was compared with group B, Ly + , V + and PL + were frequent (*p* < 0.001, all). When group D was compared with group C, the rates of male, smoker, Ly + , V + and EGFR mutation were significantly different (*p* < 0.001, all). The rate of positive EGFR mutation rate in group D was the least (13.6%) and was significantly different from group A, B and C (*p* < 0.001, all). Cases of adjuvant therapy were rare in group A.

**Table 3:** Patient information for predominant histological subtypes in pathological stage I

		Total	Group A (AIS + MIA)	Group B (Lepidic)	Group C (Papillary + acinar + variant)	Group D (Solid + micropapillary)	*p*-value
n		2165	304	469	1242	150	
Sex (male/Female), n (%)		1,058/1,107	119/185 (39.1%)	199/270 (42.4%)	635/608 (51.1%)	98/52 (65.3%)	< 0.001
Age, mean ± SD		66.76 ± 9.02	66.49 ± 9.67	67. 65 ± 8.18	66.55 ± 9.13	66.19 ± 9.23	0.201
Smoking history ( ±), n (%)		1046/1060 (49.7%)	110/187 (37.0%)	188/269 (41.1%)	642/563 (53.3%)	106/41 (72.1%)	< 0.001
Operation time (min.), mean ± SD		201.1 ± 64.30	203.47 ± 62.70	195.27 ± 64.24	203.56 ± 65.35	194.17 ± 57.46	0.028
Invasion, n (%)							
	Lymphatic vessel invasion ( ±)	371/1566 (19.2%)	0/265 (0.0%)	52/383 (12.0%)	277/823 (25.2%)	42/95 (30.7%)	< 0.001
	Vascular invasion ( ±)	350/1593 (18.0%)	0/267 (0.0%)	36/400 (8.3%)	248/853 (22.5%)	66/73 (47.5%)	< 0.001
	Pleural invasion ( ±)	383 /1770 (17.8%)	0/301 (0.0%)	51/416 (10.9%)	291/944 (23.6%)	41/109 (27.3%)	< 0.001
EGFR mutation ( ±)		459/498 (48.0%)	64/42 (60.4%)	102/74 (58.0%)	284/325 (46.6%)	9/57 (13.6%)	< 0.001
Adjuvant therapy ( ±)		563/1587 (26.2%)	24/278 (7.9%)	116/349 (24.9%)	378/857 (30.6%)	45/103 (30.4%)	< 0.001
Pathological stage IA/IB		1431/734 (66.1%)	281/23 (92.4%)	319/150 (68.0%)	749/493 (60.3%)	82/68 (54.7%)	< 0.001

Table 4 shows the characteristics of patients with the predominant subtype group in pathological stage II + IIIA. In group D, Ly + , V + and PL + were most frequent, similar to stage I. In the group C, the rates of these three factors were higher compared with group B (*p* = 0.023, *p* < 0.001, *p* = 0.003). In the group D, the rates of Ly + and V + were higher compared with group C (*p* = 0.023 v. 0.003). The rate of positive EGFR mutation in group D was also least (27.0%) and was significantly lower than group B and C (*p* = 0.030, *p* = 0.009). Two-thirds of the patients in the stage II + IIIA group received adjuvant chemotherapy.

**Table 4:** Patient information for predominant histological subtypes in pathological stage II + IIIA

		Total	Group B (Lepidic)	Group C (Papillary + acinar + variant)	Group D (Solid + micropapillary)	*p*-value
n		697	57	503	137	
Sex (male/female), n (%)		426/271 (61.1%)	34/23 (58.8%)	305/198 (60.8%)	87/50 (63.5%)	0.905
Age, mean ± SD		66.57 ± 9.53	68.00 ± 8.35	66.71 ± 9.69	65.41 ± 9.45	0.223
Smoking history ( ±), n (%)		424/248 (63.2%)	34/23 (58.8%)	291/194 (60.6%)	99/31 (76.2%)	0.003
Operation time (min.), mean ± SD		217.89 ± 72.68	219.02 ± 76.10	219.27 ± 72.75	213.19 ± 71.95	0.711
Invasion, n (%)						
	Lymphatic vessel invasion ( ±)	406/219 (65.0%)	24 /29 (45.2%)	293/155 (65.4%)	89/35 (71.8%)	< 0.001
	Vascular invasion ( ±)	345/282 (55.1%)	13/40 (26.5%)	246/206 (54.4%)	86/38 (70.5%)	< 0.001
	Pleural invasion ( ±)	324/396 (45.0%)	12/45 (23.5%)	226/275 (45.1%)	70/65 (51.9%)	< 0.001
EGFR mutation ( ±)		182/284 (39.5%)	16/22 (44.1%)	142/197 (41.9%)	24/65 (27.0%)	0.048
Adjuvant therapy ( ±)		453/233 (66.0%)	36/19 (63.3%)	319/177 (64.3%)	98/37 (72.6%)	0.249
Pathological stage II / IIIA		375/322 (53.8%)	25/32 (43.9%)	270/233 (53.7%)	73/64 (53.3%)	0.960

*AIS* Adenocarcinoma in situ, *MIA* Minimally invasive adenocarcinoma, *SD* Standard deviation

### Clinicopathological factors may influence RFS and OS.

Table 5 shows clinicopathological factors which may influence RFS. The multivariate analysis showed that age, gender, operative time, Ly + , V + , group C, group D, adjuvant chemotherapy, clinical stage IIB, IIIA and pathological stage were significant factors on RFS. In OS, the multivariate analysis showed that age, operative time, V + , group D, EGFR status, adjuvant chemotherapy, pathological stage were significant factors (Table 6). Group D had independent negative impact to both of RFS and OS.

**Table 5:** Statistical analysis of patient characteristics of RFS data

		Univariate analysis		Multivariate analysis	
		*p*–value	HR [95%CI]	*p*–value	HR [95%CI]
Age		0.007	1.011 [1.003–1.019]	0.006	1.012 [1.004–1.021]
Gender (female vs. male)		< 0.001	0.632 [0.550–0.726]	0.038	0.806 [0.657–0.988]
Smoking history (+ vs. –)		< 0.001	1.519 [1.319–1.748]	0.552	0.938 [0.761–1.157]
Operation time		< 0.001	1.003 [1.002–1.004]	< 0.001	1.002 [1.001–1.003]
Invasion					
	Lymphatic vessel invasion (+ vs. –)	< 0.001	3.123 [2.706–3.605]	0.002	1.330 [1.113–1.590]
	Vascular invasion (+ vs. –)	< 0.001	3.206 [2.779–3.698]	< 0.001	1.377 [1.155–1.643]
	Pleural invasion (+ vs. –)	< 0.001	2.574 [2.243–2.954]	0.448	1.070 [0.899–1.274]
Adenocarcinoma predominance					
AIS + MIA			control		control
Lepidic		0.01	1.694 [1.132 – 2.533]	0.678	1.100 [0.702 – 1.722]
Acinar + Papillary + Variants		< 0.001	3.952 [2.783–5.611]	0.012	1.686 [1.124 – 2.530]
Solid + Micropapillary		< 0.001	7.041 [4.820–10.285]	< 0.001	2.221 [1.421—3.472]
EGFR status (+ vs. –)		0.205	0.902 [0.768–1.058]		
Adjuvant chemotherapy (+ vs. –)		< 0.001	0.481[0.420—0.537]	0.016	0.812 [0.686—0.962]
Clinical stage					
	Stage IA		control		control
	Stage IB	< 0.001	1.802 [1.535—2.115]	0.332	1.099 [0.908—1.329]
	Stage IIA	< 0.001	3.097 [2.481—3.866]	0.19	1.191 [0.917—1.546]
	Stage IIB	< 0.001	3.916 [2.913—5.266]	0.024	1.500 [1.055—2.135]
	Stage IIIA	< 0.001	5.774 [4.505—7.401]	0.017	1.454 [1.069—1.978]
Pathological stage					
	Stage IA		control		control
	Stage IB	< 0.001	2.556 [2.097—3.116]	< 0.001	1.888 [1.459—2.442]
	Stage IIA	< 0.001	5.767 [4.621—7.197]	< 0.001	3.482 [2.613—4.640]
	Stage IIB	< 0.001	7.042 [5.316—9.328]	< 0.001	3.953 [2.737—5.710]
	Stage IIIA	< 0.001	10.288 [8.462—12.508	< 0.001	5.681 [4.247—7.600]

**Table 6:** Statistical analysis of patient characteristics of OS data

		Univariate analysis		Multivariate analysis	
		*p*-value	HR [95%CI]	*p*-value	HR [95%CI]
Age		< 0.001	1.038 [ 1.027–1.049]	< 0.001	1.032 [1.017—1.048]
Gender (female vs. male)		< 0.001	0.491 [0.409–0.591]	0.447	0.884 [0.643—1.215]
Smoking History (+ vs. –)		< 0.001	2.108 [1.741–2.554]	0.172	1.265 [0.903—1.771]
Operation time		< 0.001	1.003 [1.002–1.004]	0.016	1.002 [1.001–1.004]
Invasion					
	Lymphatic vessel invasion (+ vs. –)	< 0.001	3.056 [2.533–3.685]	0.378	1.140 [0.852—1.525]
	Vascular invasion (+ vs. –)	< 0.001	3.125 [2.597–3.759]	0.015	1.417 [1.071—1.874]
	Pleural invasion (+ vs. –)	< 0.001	2.519 [2.113–3.004]	0.518	1.096 [0.830—1.447]
Adenocarcinoma predominance					
AIS + MIA			control		control
Lepidic		0.198	1.360[0.852–2.172]	0.998	0.999 [0.455—2.197]
Acinar + Papillary + Variants		< .001	2.600 [1.745–3.876]	0.380	1.366 [0.681—2.739]
Solid + Micropapillary		< .001	5.178 [3.355–7.991]	0.041	2.182 [1.031—4.617]
EGFR status (+ vs. –)		< 0.001	0.551 [0.439–0.692]	0.050	0.762 [0.581—1.000]
Adjuvant chemotherapy (+ vs. –)		< 0.001	0.645 [0.769–0.541]	0.034	0.740 [0.561—0.977]
Clinical stage					
	Stage IA		control		control
	Stage IB	< 0.001	1.778[1.445—2.188]	0.796	1.041 [0.767—1.414]
	Stage IIA	< 0.001	3.032 [2.295—4.005]	0.358	1.204 [0.810—1.790]
	Stage IIB	< 0.001	2.853 [1.911—4.259]	0.701	0.882 [0.466—1.671]
	Stage IIIA	< 0.001	4.581 [3.347—6.270]	0.938	0.981 [0.602—1.597]
Pathological stage					
	Stage IA		control		control
	Stage IB	< 0.001	2.038 [1.578—2.631]	0.026	1.640 [1.062—2.534]
	Stage IIA	< 0.001	4.513 [3.419—5.957]	< 0.001	2.588 [1.622—4.131]
	Stage IIB	< 0.001	4.961 [3.440—7.154]	0.027	2.063 [1.087—3.915]
	Stage IIIA	< 0.001	6.695 [5.244—8.548]	< 0.001	4.112 [2.559—6.606]

*HR* Hazard ratio

## Discussion

To our knowledge, this is the most expansive report to date on the relationship of prognostic outcome and lung adenocarcinoma predominant subtype. By selecting cases who had undergone lobectomy with mediastinal lymph-node dissection, it enabled us to minimize stage migration, and figure out precise malignant behavior of each predominant subtype. As expected, patients with group A showed excellent outcome. Majority of them were in stage I, with only 3.0% in a higher stage. However, even in the group A, there was a small percentage with lymph node metastasis. Especially in MIA, defined as invasion of size ≤ 5 mm, if an aggressive component exists in this small invasive lesion, it might metastasize to the hilar and/or mediastinal lymph node and up staged.

From the report limited to stage I, invasive adenocarcinoma has been further grouped as intermediate or high grade [4]. In the intermediate grade group of lepidic, acinar and papillary, lepidic tends to show better prognoses than other [4]. Actually, in this study, lepidic showed the second-better outcome. The 5-year RFS and OS rates for lepidic were nearly the same as for group A; however, the rate of stage II + IIIA in lepidic was 10.8%, three times higher than group A. This indicates that the malignant activity of lepidic was not same good as group A. Lepidic component is well differentiated lung adenocarcinoma, its malignant potential is considered to be low [3]. But lepidic predominant tumor sometimes contains other more malignant subtypes. If the ratio of those other malignant component is high, its prognosis will naturally be worse than more lepidic dominant tumor. The 5-year RFS and OS rates of acinar, papillary and variants were similar and so these subtypes can be dealt with as the same. In previous reports, prognostic outcome of these acinar and papillary predominant was reported as similar, we reconfirmed it again [3, 4, 7]. For variants, there have been few reports about the surgical prognostic outcome [15] which question how to estimate variants’ malignant activity. From the prognostic outcome, we conclude that variants should be categorized as same of papillary and acinar predominant. This group showed the second worse 5-year RFS and OS rates and better than the group D; solid + micropapillary. But in advanced stage, the prognostic impact of these predominant; lepidic, acinar, papillary and variants did not differ. They should be dealt separately whether in case of stage I or II + IIIA.

The 5-year RFS and OS rate of solid and micropapillary predominant were similar, thus, these subtypes can be dealt as one group. Group D showed the worst outcome, not only in early stage but also in higher stage. Originally, in pathological stage I, these subtypes were categorized as high-grade. But even in higher pathological stages of II + IIIA, it also showed worst outcome than other subtype groups. Up stage rate of group D from clinical stage I to pathological stage II + IIIA was 33.8%, much higher than other groups. It also indicates potential malignant behavior of group D. Multivariate analysis including pathological stage showed independent negative impact of group D for both RFS and OS rates.

We examined pathological invasive factors to analyze negative impact to RFS and OS rates in each predominance groups. Three factors; Ly + , V + and PL + were reported as showing significantly worse prognosis in lung cancer [16, 17]. In this study, two pathological invasive factors of Ly + and V + were reconfirmed as independent prognostic factors. They were scarce in group A and B, frequent in group C, and more frequent in group D. Although lepidic; group B was categorized as invasive adenocarcinoma, the better outcome in early stage was explained by less frequency of these factors. The tendency of more frequent invasive factors in group D was observed not only in stage I but also in stage II + IIIA. These differences may reflect the prognostic outcome of each predominance groups, especially in group D. The reason that the worst OS rate of group D was observed in early and locally advanced stage, might be related to the rate of EGFR mutation. It is well known that positive EGFR mutation shows higher response to EGFR tyrosine kinase inhibitors (TKI) than EGFR wild type and can improve OS rate even after recurrence [18–21]. Least frequency of EGFR mutation in group D compared with other predominant groups reduces the treatment chance using EGFR-TKI after recurrence, it may reflect poorer OS outcome.

The IASLC/ATS/ERS classifications was introduced in 2011, they separated invasive adenocarcinoma into 5 types according to dominant histologic pattern by microscopic findings. Including AIS, MIA and variants, there are totally 8 types of predominance. This classification was corelated with prognosis, but the number as a prognostic indicator thought to be too many, it would be inconvenient in clinical situation. And small percent of solid or micropapillary component regardless of its main predominance was reported to have negative impact for prognosis [12]. In this meaning, it would be time to be re-categorized into smaller numbers according to prognostic impact. Actually, in other organs, tumor grading system including biomarkers had been introduced to reflect its prognostic outcome [22–26]. In lung cancer, a new modified grading system based on these traditional predominant subtypes has just proposed [27]. The validity of this system should be examined with large scale database near future.

There are several limitations in this study. First, although this is an expansive report on the analysis of the impact of predominant histological subtype of adenocarcinoma, it is a retrospective study and potential bias may exist. Second, central review of pathological factors including histology of adenocarcinoma, lymphatic vessel invasion, vascular invasion and pleural invasion was not performed. Third, EGFR status was only known for half of cases included. Most of them was examined in recurred cases, it might not reflect true characteristics of each pathological predominant group. Fourth, postoperative surveillance was not uniform and might have affected the time of detecting recurrence.

## Conclusion

Predominant subtype of adenocarcinoma is one of the prognostic factors. Especially, solid + micropapillary showed the worst outcome not only in stage I but also in stage II + IIIA. The frequency of pathological invasive factors and EGFR mutation might be related to the malignant behavior of each adenocarcinoma predominance.

## Data Availability

The data that support the findings of this study are available from the Japanese Joint Committee of Lung Cancer Registry Database but restrictions apply to the availability of these data, which were used under license for the current study, and so are not publicly available. Data are however available from the authors upon reasonable request and with permission of the Japanese Joint Committee of Lung Cancer Registry Database.
